# Risk factors for mortality and development of a predictive model in pediatric sepsis

**DOI:** 10.3389/fped.2025.1661086

**Published:** 2025-09-22

**Authors:** Caizhen Wang, Lijie Feng, Xiaohui Yang, Pan Wang, Yuan Chen

**Affiliations:** Pediatric Intensive Care Unit, The Second Hospital of Hebei Medical University, Shijiazhuang, China

**Keywords:** children, sepsis, mortality risk factors, CALLY index, albumin

## Abstract

**Objective:**

To investigate the clinical characteristics and risk factors associated with mortality in pediatric sepsis patients, and to establish a predictive model for early identification of high-risk children.

**Methods:**

A retrospective cohort study was conducted including 143 pediatric sepsis cases admitted to the Pediatric Intensive Care Unit of the Second Hospital of Hebei Medical University from January 2020 to December 2024. Clinical data, laboratory indicators, and treatment history were collected. Univariate and multivariate logistic regression analyses were performed to identify risk factors for mortality. A nomogram model was constructed based on significant predictors, and its predictive performance was evaluated by receiver operating characteristic (ROC) curve analysis.

**Results:**

Among the 143 cases, 121 survived and 22 died. Significant differences were observed between the survival and death groups in lymphocyte count, platelet count, albumin, D-dimer, liver function tests (ALT, TBIL), CALLY index, and pre-admission glucocorticoid use (*P* < 0.05). Multivariate analysis identified platelet count (OR = 0.992, 95% CI: 0.987–0.997), D-dimer (OR = 7.571, 95% CI: 2.642–21.698), and CALLY index (OR = 0.532, 95% CI: 0.323–0.877) as independent risk factors for mortality. The nomogram model incorporating these factors showed good predictive accuracy with an area under the ROC curve of 0.859 (95% CI = 0.742–0.953).

**Conclusion:**

Platelet count, D-dimer level, and CALLY index are valuable indicators for assessing prognosis in pediatric sepsis and can aid in early risk stratification. The established nomogram provides a useful tool for clinical decision-making to improve outcomes in high-risk pediatric sepsis patients. Further multicenter prospective studies are warranted to validate and refine these findings.

## 1 Introduction

Sepsis is a life-threatening organ dysfunction caused by a dysregulated host immune response to infection and has become one of the leading causes of mortality among pediatric patients in pediatric intensive care units (PICUs) worldwide (1, 2). Despite significant advances in anti-infective therapies and intensive care technologies in recent years, the incidence and mortality rates of pediatric sepsis remain unacceptably high (3). The disease is characterized by high clinical heterogeneity and rapid progression, posing major challenges for early diagnosis and prognostic evaluation (4, 5). Therefore, timely identification of high-risk children with sepsis at an early stage of the disease has become a critical issue for clinicians.

The C-reactive protein–albumin–lymphocyte (CALLY) index is a novel composite biomarker that integrates three routinely measured laboratory parameters—C-reactive protein (CRP), serum albumin, and absolute lymphocyte count—into a single score, calculated as (lymphocyte count × albumin)/CRP. This index simultaneously reflects systemic inflammation, nutritional and hepatic status, and immune competence, all of which are closely linked to sepsis pathophysiology and prognosis (6). Originally proposed and validated in adult populations with liver disease, cancer, and systemic inflammatory conditions, the CALLY index has recently been investigated as a prognostic marker in sepsis, showing promising predictive value for mortality and adverse outcomes (7–9). However, its applicability in pediatric sepsis remains largely unexplored. Given that children with sepsis often present with unique immune responses and disease trajectories compared to adults, we hypothesized that the CALLY index might serve as a practical and readily obtainable tool for early risk stratification in pediatric patients. Therefore, we incorporated the CALLY index into our analysis to evaluate its prognostic performance in this population.

In this study, we conducted a retrospective cohort analysis of 143 pediatric sepsis patients admitted to the Pediatric Intensive Care Unit of the Second Hospital of Hebei Medical University between January 2020 and December 2024. Based on the international diagnostic criteria for pediatric sepsis (10), we systematically summarized the clinical profile of these patients and identified independent risk factors significantly associated with mortality through multivariate regression analysis. The findings of this study aim to provide an objective and quantitative basis for the early identification of high-risk pediatric sepsis cases in clinical practice. We hypothesized that certain laboratory parameters, particularly the CALLY index, platelet count, and D-dimer level, are independently associated with mortality in pediatric sepsis and can be used to develop a predictive model for early risk stratification.

## 2 Methods

### 2.1 Study population

This study adopted a retrospective cohort design and included 143 pediatric patients diagnosed with sepsis who were admitted to the PICU of the Second Hospital of Hebei Medical University between January 2020 and December 2024.

Inclusion criteria: (1) Age between 28 days and 14 years. This age range was chosen because, in our institution, patients older than 14 years are generally admitted to the adult ICU, while neonates (≤28 days) are admitted to the Neonatal Intensive Care Unit (NICU) for specialized management; (2) Complete clinical medical records; (3) Diagnosis consistent with the 2012 international pediatric sepsis criteria (6).

Exclusion criteria: (1) Age >14 years or ≤28 days; (2) Incomplete clinical data; (3) Length of hospital stay <24 h; (4) For patients with multiple hospitalizations due to sepsis, only the first admission was included for analysis.

Patients were divided into the survival group and the non-survival group based on their discharge outcomes.

This study was approved by the Ethics Committee of the Second Hospital of Hebei Medical University (Approval No. 2024-R253).

### 2.2 Data collection

The following clinical data were extracted from the hospital's electronic medical record system: (1) Baseline characteristics: age, sex, and comorbidities (e.g., hematologic malignancies, congenital anomalies); (2) Treatment-related factors: history of glucocorticoid use within 30 days prior to admission, central venous catheterization, mechanical ventilation, and other interventions; (3) Laboratory parameters: inflammatory markers (WBC, CRP, PCT) measured within 24 h of admission; immune function indicators (lymphocyte subsets, immunoglobulins); coagulation parameters (D-dimer, APTT, PT); liver and kidney function tests (ALT, AST, creatinine); and blood culture results.

If a parameter was measured multiple times within the first 24 h, the value closest to the time of PICU admission was recorded to best reflect the patient's baseline status before the influence of treatment interventions. The CALLY index was calculated using the following formula: CALLY = (absolute lymphocyte count × albumin)/CRP (11).

### 2.3 Statistical analysis

Statistical analyses were performed using SPSS version 24.0 and R version 4.3.2. For continuous variables with a normal distribution, data were expressed as mean ± standard deviation and compared between groups using the t-test. For non-normally distributed continuous variables, data were presented as median and interquartile range (*IQR*), and the Mann–Whitney *U* test was used for group comparisons. Categorical variables were expressed as frequency and percentage (%), and compared using the chi-square test. Variables with statistically significant differences in univariate analysis were included in univariate and multivariate logistic regression models. The results were reported as odds ratios (ORs), 95% confidence intervals (CIs), and *P*-values. Statistically significant variables from the multivariate logistic regression were used to construct a nomogram and receiver operating characteristic (ROC) curve. To assess internal validity and minimize optimism bias, model performance was further evaluated using bootstrapping with 1,000 resamples, and the optimism-corrected AUC was calculated. All statistical tests were two-tailed, and a *P*-value < 0.05 was considered statistically significant.

## 3 Results

### 3.1 Clinical characteristics of the two groups

A total of 143 pediatric patients with sepsis were included in this study, of whom 121 survived and 22 died. Significant differences were observed between the survival and non-survival groups in terms of white blood cell count (WBC), lymphocyte count, platelet count, albumin (ALB), total bilirubin (TBIL), D-dimer, alanine aminotransferase (ALT), estimated glomerular filtration rate (eGFR), clinical severity scores (PRISM III and pSOFA), vasoactive-inotropic score (VIS), CALLY index, and prior use of glucocorticoids before hospitalization (*P* < 0.05). No statistically significant differences were found in age, sex, disease duration, neutrophil count, or PELOD-2 score between the two groups (*P* > 0.05) (Table 1).

**Table 1:** Comparison of clinical characteristics in pediatric sepsis patients.

Variable	Survival (*n* = 121)	Non-survival (*n* = 22)	t/Z/*χ*^2^	*P* value
Age (months)	59.0 (29.0,79.5)	54.0 (26.8,64.5)	−0.415	0.678
Sex (male, %)	65 (53.7)	12 (54.5)	0.005	0.943
Disease duration (days)	6.0 (3.0,8.5)	5.5 (2.8,7.3)	−0.671	0.502
Auxiliary examinations at admission				
WBC(×10^9 ^/L)	16.1 (15.0,18.3)	10.8 (7.8,13.6)	−2.784	0.005
Neutrophil count (×10^9 ^/L)	10.2 (9.0,12.0)	9.7 (8.1,12.4)	−0.719	0.472
lymphocyte count (×10^9 ^/L)	5.5 (3.8,6.6)	2.4 (1.6,4.2)	−4.928	<0.001
Platelet count (×10^9 ^/L)	420.8 (346.4,520.5)	328.0 (289.0,400.9)	−3.494	<0.001
CRP (mg/L)	97.7 (66.3,122.3)	99.3 (63.93,113.6)	−0.014	0.989
PCT (ng/ml)	7.9 (6.6,9.6)	8.7 (6.0,11.6)	0.915	0.360
Lac (mmol/L)	3.1 (2.3,4.0)	3.6 (2.9,4.6)	1.847	0.065
ALB (g/L)	45.1 (41.7,47.1)	39.9 (37.4,46.0)	−2.708	0.007
TBIL (μmol/L)	19.8 (15.0,22.2)	23.9 (17.8,42.9)	2.815	0.005
GLU (mmol/L)	9.01 ± 1.02	8.95 ± 1.13	−0.168	0.867
Cr (mol/L)	55.9 (36.8,79.9)	63.6 (37.7,70.2)	−0.330	0.741
D-Dimer (μg/ml DDU)	0.84 (0.74,0.88)	1.40 (0.94,1.94)	4.256	<0.001
APTT (s)	36.12 ± 6.06	35.60 ± 5.75	0.370	0.μ712
PT (s)	11.63 ± 1.13	11.72 ± 1.15	−0.327	0.744
ALT (U/L)	76.8 (37.9,131.8)	121.6 (72.3,168.9)	2.199	0.028
AST (U/L)	69.2 (32.1,97.7)	69.1 (38.2,69.5)	−0.588	0.557
IgG (g/L)	11.93 ± 2.79	11.53 ± 2.67	0.634	0.527
IgM (g/L)	1.7 (1.2,2.1)	1.7 (1.1,2.1)	−0.081	0.935
IgA (g/L)	2.5 (1.5,3.2)	2.6 (1.4,3.3)	0.076	0.940
eGFR (ml/min/1.73 m^2^)	140.0 (90.5,181.5)	90.0 (64.5,132.5)	−2.638	0.008
CALLY index	2.4 (1.6,3.7)	1.2 (0.6,2.0)	−3.961	<0.001
Clinical score				
PRISM III	8.0 (6.0,10.0)	10.0 (8.75,11.0)	2.995	0.003
PELOD 2	4.0 (3.0,5.0)	5.0 (3.0,6.0)	1.679	0.093
pSOFA	5.0 (4.0,7.0)	6.0 (5.0,8.0)	2.385	0.017
Peak VIS 24 h	7.65 (4.41,11.08)	10.68 (6.45,14.63)	2.325	0.020
Pre-existing condition				
Hematopoietic malignancies	15 (12.4)	6 (27.3)	3.288	0.070
Diabetes mellitus	22 (18.2)	3 (13.6)	0.267	0.606
Congenital malformations	4 (3.3)	2 (9.1)	1.550	0.213
Pre-infection related treatment				
Glucocorticoid therapy	27 (22.3)	17 (77.3)	26.395	<0.001
Central venous catheterization	12 (9.9)	5 (22.7)	2.916	0.088
Mechanical ventilation	9(7.4)	3(13.6)	0.930	0.335

WBC, white blood cell; CRP, C-reactive protein; PCT, procalcitonin; Lac, serum lactate; ALB, serum albumin; TBIL, total bilirubin; GLU, blood glucose; Cr, creatinine; APTT, activated partial thromboplastin time; PT, prothrombin time; ALT, alanine aminotransferase; AST, aspartate aminotransferase; IgG, immunoglobulin G; IgM, immunoglobulin M; IgA, immunoglobulin A; CALLY index, C-reactive protein-albumin-lymphocyte index. Data are presented as mean ± standard deviation (SD) for normally distributed continuous variables and as median (interquartile range, IQR) for non-normally distributed continuous variables. Categorical variables are expressed as number (percentage).

### 3.2 Risk factor analysis for mortality in pediatric sepsis patients

Univariate logistic regression analysis was performed for WBC, lymphocyte count, platelet count, ALB, TBIL, D-dimer, ALT, and CALLY index. The results indicated that WBC, lymphocyte count, platelet count, ALB, D-dimer, ALT, and CALLY index were significantly associated with mortality in pediatric sepsis patients (Table 2).

**Table 2:** Univariate analysis of risk factors for mortality in pediatric sepsis patients.

Variable	OR	95% CI	*P* value
WBC(×10^9 ^/L)	0.801	0.698–0.920	0.002
lymphocyte count (×10^9 ^/L)	0.475	0.343–0.656	<0.001
Platelet count (×10^9 ^/L)	0.994	0.990–0.998	0.003
ALB (g/L)	0.846	0.756–0.946	0.003
TBIL (μmol/L)	1.019	0.998–1.040	0.079
D-Dimer (μg/ml DDU)	6.529	2.074–20.554	0.001
ALT (U/L)	1.010	1.001–1.018	0.025
CALLY index	0.470	0.287–0.771	0.003

Considering the strong correlations of WBC, lymphocyte count, ALB, and ALT with the CALLY index, only variables with statistical significance in univariate analysis were included in the multivariate logistic regression model. The results showed that platelet count (OR = 0.992, 95% CI: 0.987–0.997), D-dimer (OR = 7.571, 95% CI: 2.642–21.698), and CALLY index (OR = 0.532, 95% CI: 0.323–0.877) were independent risk factors for mortality in pediatric sepsis patients (Table 3).

**Table 3:** Multivariate analysis of risk factors for mortality in pediatric sepsis patients.

Variable	β	SE	Wald	*p*	OR	95% CI
Platelet count (×10^9 ^/L)	−0.008	0.003	9.066	0.003	0.992	0.987–0.997
D-Dimer (μg/ml DDU)	2.024	0.537	14.202	<0.001	7.571	2.642–21.698
CALLY index	−0.631	0.255	6.132	0.013	0.532	0.323–0.877

### 3.3 Development of a mortality risk model for pediatric sepsis patients

**Figure 1:**
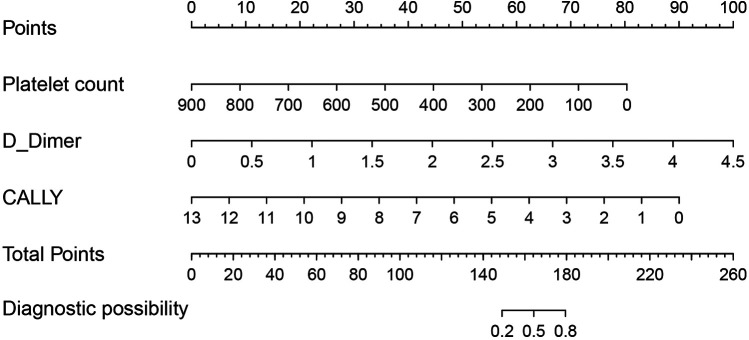
Nomogram for predicting mortality risk in pediatric sepsis patients.

**Figure 2:**
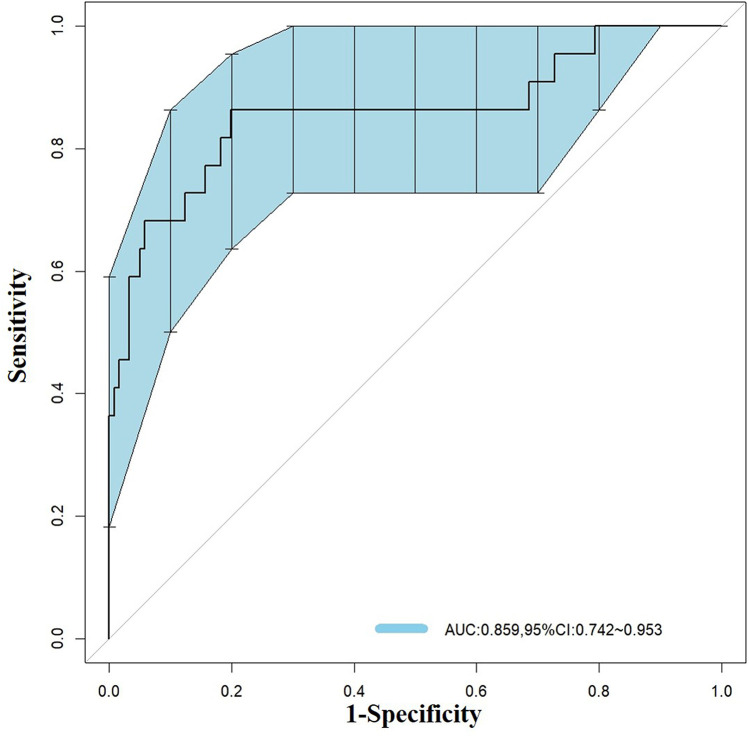
ROC curve for predicting mortality risk in pediatric sepsis patients.

Based on the three factors identified by multivariate logistic regression analysis, a nomogram model was constructed (Figure 1). The total score, obtained by summing the points assigned to each predictive factor, corresponds to the probability of mortality risk in pediatric sepsis patients. The predictive performance of the nomogram model was evaluated using the ROC curve, which showed an area under the curve (AUC) of 0.859 (95% CI = 0.742–0.953), indicating good predictive accuracy (Figure 2).

## 4 Discussion

This study retrospectively analyzed the clinical data of 143 pediatric sepsis patients admitted to the Pediatric Intensive Care Unit of Hebei Medical University Second Hospital, comparing multiple clinical indicators between the survival and non-survival groups. Significant differences were observed in peripheral blood white blood cell count, lymphocyte count, platelet count, albumin, total bilirubin, D-dimer, ALT, CALLY index, and prior use of glucocorticoids before hospitalization. These findings suggest that these indicators may be closely associated with the prognosis of pediatric sepsis patients. Notably, patients in the non-survival group generally exhibited lower lymphocyte counts, platelet counts, and albumin levels, alongside higher levels of D-dimer and total bilirubin, reflecting more severe immunosuppression, coagulation dysfunction, and liver injury. The CALLY index, as a novel composite indicator reflecting nutritional and hepatic status, also demonstrated a certain discriminative ability in this study (11–13). Further regression analyses and the mortality risk prediction model indicated that platelet count, D-dimer level, and CALLY index could serve as important early reference markers for identifying high-risk pediatric sepsis patients, providing strong support for clinical risk assessment and early intervention.

The results of this study are consistent with previous research on both pediatric and adult sepsis in several aspects (14, 15). The more pronounced decrease in lymphocyte count observed in the non-survival group aligns with studies highlighting immunosuppression during severe infections (16, 17). Demonstrated that increased lymphocyte apoptosis and impaired T cell function in septic children are key mechanisms contributing to immune dysregulation and elevated mortality (18, 19). Additionally, the significantly elevated D-dimer levels in the non-survival group suggest the presence of coagulation dysfunction, which is consistent with findings in adult sepsis where D-dimer serves as an independent risk factor for poor prognosis (20, 21). This study also found that albumin levels were significantly lower in the non-survival group compared to the survival group, indicating its potential role as an early warning marker reflecting nutritional status and systemic inflammation, which aligns with multiple reports identifying hypoalbuminemia as a prognostic indicator of adverse outcomes in sepsis (22, 23). In addition to being a marker of chronic nutritional deficiency, hypoalbuminemia in sepsis also reflects endothelial dysfunction, vascular hyperpermeability, and capillary leak, all of which contribute to intravascular volume depletion, tissue edema, and organ dysfunction (24). These mechanisms may partly explain the strong association between low albumin levels and poor prognosis in septic patients.

Notably, this study also found a higher proportion of glucocorticoid use prior to hospitalization in the non-survival group, which may be related to their more severe underlying diseases and more complex immune status. Although glucocorticoids exhibit anti-inflammatory effects in certain severe infections, previous studies have indicated that inappropriate use may delay infection control and increase the risk of secondary infections, thereby adversely affecting prognosis (25, 26). Additionally, elevated liver function indicators such as ALT and TBIL suggest that the septic process may involve hepatic injury, reflecting a trend toward multi-organ dysfunction, which has also been reported in previous studies (27).

In this study, a lower CALLY index was significantly associated with increased mortality among pediatric patients with sepsis, highlighting its potential role as a prognostic biomarker in this population. The CALLY index, which integrates serum albumin, lymphocyte count, and C-reactive protein levels, provides a composite reflection of nutritional status, immune competence, and systemic inflammation—three key elements intricately linked to sepsis progression and outcomes (7, 28). Although originally developed and validated in adult populations with liver disease and systemic inflammatory conditions, our findings suggest that the CALLY index may also have meaningful clinical applicability in pediatric sepsis. Its ability to synthesize multiple clinically relevant parameters into a single, easily obtainable index makes it a practical tool for early risk stratification and outcome prediction. As such, the incorporation of the CALLY index into pediatric sepsis assessment protocols could assist clinicians in identifying high-risk patients more efficiently and guiding timely intervention strategies. Further multicenter and prospective studies are warranted to validate its utility and optimize its thresholds in the pediatric setting.

The findings of this study have important implications for clinical practice. By identifying key clinical indicators that affect the prognosis of pediatric sepsis—such as lymphocyte count, platelet count, albumin, D-dimer, and liver function-related markers—clinicians can perform early risk stratification upon admission. This enables more targeted monitoring and intervention. Especially in cases of pediatric sepsis with rapid disease progression and atypical symptoms, integrating these laboratory parameters into a comprehensive assessment can improve the sensitivity of early detection, shorten diagnostic time, and optimize treatment strategies (29). The CALLY index, as an easily obtainable composite biochemical index, demonstrated predictive value in this study, indicating its potential utility in risk assessment for pediatric sepsis and warranting further clinical validation and broader application (30).

In our PICU, immunoglobulin levels are routinely measured in all patients with suspected sepsis as part of the initial laboratory evaluation. In the present study, these parameters did not differ significantly between survivors and non-survivors. This may indicate that humoral immunity, as reflected by immunoglobulin concentrations, remains relatively preserved in pediatric sepsis, whereas cellular immunity—particularly T-lymphocyte-mediated responses—may be more significantly impaired. The pronounced lymphopenia observed in the non-survival group supports this possibility. Previous studies have shown that sepsis can induce profound *T*-cell depletion, functional exhaustion, and apoptosis, leading to sustained immunosuppression and worse outcomes (31). Therefore, further investigation into T-lymphocyte dynamics and function in pediatric sepsis could provide valuable insights into disease pathophysiology and guide targeted immunomodulatory therapies.

This study suggests that pre-admission use of glucocorticoids may be associated with poor prognosis, indicating that clinicians should exercise greater caution when administering glucocorticoids, especially in cases where infection control status or disease stage is not clearly established. The potential benefits and risks should be carefully evaluated (32, 33). Overall, identifying these quantifiable laboratory indicators not only aids in enhancing early recognition and intervention for critically ill pediatric patients but also provides foundational data to support the development of prognostic models for pediatric sepsis and to advance precision medicine. In the future, integrating these findings into clinical pathways or scoring systems may help improve overall diagnostic and treatment quality and reduce sepsis-related mortality in children.

Although this study systematically analyzed the clinical characteristics and prognostic indicators of pediatric sepsis, providing certain clinical guidance, several limitations remain. First, as a single-center retrospective study with a relatively small sample size, selection bias may be present. Second, this study only included indicators measured during hospitalization and lacked follow-up data on long-term outcomes such as readmission rates and neurological sequelae, which limits the scope of assessing the long-term prognosis of sepsis. Therefore, the conclusions need to be further validated in larger, multicenter prospective studies. In addition, because this was a retrospective analysis, no *a priori* power calculation was performed. The relatively small number of mortality events means that the sample size may be borderline for the recommended “events per variable” threshold in multivariable logistic regression, which could affect the stability of the model estimates and limit the generalizability of our findings. Nonetheless, this study offers valuable insights for the early assessment and risk identification of pediatric sepsis and lays a foundation for future mechanistic research and clinical translation.

The predictive model developed in this study—incorporating platelet count, D-dimer, and CALLY index—has the potential to facilitate early risk stratification in pediatric sepsis, enabling clinicians to identify patients at highest risk of mortality during the initial stage of PICU admission. Early recognition could allow for closer hemodynamic and laboratory monitoring, more rapid escalation of antimicrobial and organ support therapies, and consideration of adjunctive treatments such as intravenous immunoglobulin (IVIG) or plasma exchange in selected patients. While these aggressive interventions have shown potential benefits in certain adult and pediatric septic populations, robust evidence in the pediatric setting remains limited. Therefore, future multicenter prospective studies should explore whether targeted early application of such therapies, guided by validated risk prediction models, can improve survival and long-term outcomes in high-risk children with sepsis.

## 5 Conclusion

This study retrospectively analyzed 143 hospitalized pediatric sepsis cases and found that the deceased group showed significant differences compared to the survival group in lymphocyte count, platelet count, albumin, D-dimer, liver function indicators (ALT, TBIL), CALLY index, and pre-hospital use of glucocorticoids. These indicators can serve as important references for assessing the severity and prognosis of pediatric sepsis. In particular, platelet count, CALLY index, and D-dimer level demonstrated good clinical predictive value and hold potential for early identification of high-risk patients and guiding intervention decisions. Despite certain limitations, the findings provide valuable insights for early risk assessment and personalized management of pediatric sepsis in clinical practice. Future studies with larger sample sizes and multicenter prospective designs are needed to further validate these results and to explore additional biomarkers and scoring systems for early warning, aiming to improve the overall diagnosis, treatment, and survival rates of pediatric sepsis.

## Data Availability

The raw data supporting the conclusions of this article will be made available by the authors, without undue reservation.
